# Fecal fermentation characteristics of *Rheum tanguticum* polysaccharide and its effect on the modulation of gut microbial composition

**DOI:** 10.1186/s13020-022-00631-6

**Published:** 2022-06-23

**Authors:** Ding-Tao Wu, Qin Yuan, Kang-Lin Feng, Jinming Zhang, Ren-You Gan, Liang Zou, Shengpeng Wang

**Affiliations:** 1 Key Laboratory of Coarse Cereal Processing, Ministry of Agriculture and Rural Affairs, Sichuan Engineering & Technology Research Center of Coarse Cereal Industralization, School of Food and Biological Engineering, Chengdu University, Chengdu, 610106 China; 2 State Key Laboratory of Quality Research in Chinese Medicine, Institute of Chinese Medical Sciences, University of Macau, Macao, China; 3 State Key Laboratory of Southwestern Chinese Medicine Resources, School of Pharmacy, Chengdu University of Traditional Chinese Medicine, Chengdu, 611137 China; 4 Research Center for Plants and Human Health, Institute of Urban Agriculture, Chinese Academy of Agricultural Sciences, Chengdu, 610213 China

**Keywords:** *Rheum tanguticum*, Polysaccharide, Digestive stability, Fermentation characteristic, Microbial composition

## Abstract

**Background:**

*Rheum tanguticum* is utilized as one of the well known traditional Chinese medicine for the treatment of gastrointestinal diseases. Recently, *R. tanguticum* polysaccharides (RP) have received increasing attention due to their diversely pharmacological activities. Usually, the pharmacological activities of polysaccharides are closely correlated to their metabolic properties from the stomach to the intestine. However, the digestive behavior and fecal fermentation characteristics of RP are unknown, which need to be fully investigated.

**Methods:**

In this study, an in vitro simulated gastrointestinal model was carried out for the investigation of the digestive behavior and fecal fermentation characteristics of RP. The possible changes in physicochemical properties of RP, such as molecular weight, monosaccharide composition, reducing sugar released, chemical composition, pH value, and short chain fatty acids, were determined during in vitro simulated digestion and human fecal fermentation, and its effect on the modulation of gut microbial composition was also evaluated.

**Results:**

The results revealed that RP was indigestible under the in vitro simulated digestion conditions according to its stabilities in physicochemical properties. Conversely, the indigestible RP (RPI) could be notably utilized by colonic microbiota in human feces after the in vitro fermentation, especially, at the initial fermentation stage (0–6 h). The fecal fermentation characteristics of RPI were revealed. Results showed that the content of reducing sugars obviously increased from 0.177 to 0.778 mg/mL at the initial stage of fermentation, and its molecular weight notably declined from 2.588 × 10^5^ to 0.828 × 10^5^ Da at the end stage of fermentation. Notably, the utilization of arabinose and galactose in RPI by colonic bacteria was faster than that of galacturonic acid. Besides, RPI could obviously modulate gut microbial composition via promoting the relative abundances of several beneficial bacteria, such as genera *Bacteroides*, *Bifidobacterium*, and *Megamonas*, resulting in the promoted production of several short-chain fatty acids, such as acetic, propionic, and butyric acids.

**Conclusions:**

Results from this study showed that RP was indigestible in the human upper gastrointestinal tract in vitro, but could be easily utilized by colonic microbiota in human feces at the initial stage of fermentation. RP could be used as potential prebiotics for the improvement of intestinal health.

## Background

*Rheum tanguticum* Maxim. ex Balf., belongs to the family of Polygonaceae, is widely cultivated in Southwest and Northwest China [1]. The roots and rhizomes of *R. tanguticum* have been utilized as well known traditional Chinese medicine for the treatment of gastrointestinal diseases [2], which have also been used as functional and healthy food ingredients in China. Generally, *R. tanguticum* contains various bioactive components, such as polysaccharides, flavonoids, anthraquinones, saponins, tannins, and volatile oils [1–4], which contribute to its multiple pharmacological activities of purgative, immunomodulatory, anti-oxidant, anti-inflammatory, anti-diabetic, anti-fungal, hepatoprotective, anti-tumor, and nephroprotective effects. Especially, *R. tanguticum* polysaccharides have received increasing attention due to its diversely pharmacological activities, such as immunomodulatory effect, ameliorating radiation-induced intestinal injury, preventing radiation-induced immune damage, and ameliorating inflammatory bowel disease [5–8].

The biological activities of natural polysaccharides are closely correlated to their metabolic properties from the stomach to the intestine. Recently, increasing studies using in vitro models indicate that non-starch polysaccharides can not be entirely digested into oligosaccharides and monosaccharides or directly absorbed in human upper gastrointestinal tract due to the absence of carbohydrate active enzymes (CAZymes), consequently, these indigestible polysaccharides can reach large intestine to be fermented [9–13]. Generally, thousands of microbial species live in human intestine, and the microbial composition is closely related to the host health. Notably, the gut microbiota can encode a large number of CAZymes for the degradation of natural polysaccharides into small sugars, further exerting beneficial effects on the host [14]. Numerous studies have proven that the indigestible polysaccharides can modulate the gut health via promoting the proliferation of some beneficial bacteria, such as *Bacteroides*, *Lactobacillus*, and *Bifidobacterium* species [9, 11, 15]. At the same time, the indigestible polysaccharides can be utilized by the relevant human gut bacteria to produce several metabolic products, such as short chain fatty acids (SCFAs, primarily acetate, propionate and butyrate), which have been implicated in a low risk of metabolic syndromes, such as obesity, dyslipidaemia, insulin resistance, and inflammatory bowel diseases [16, 17]. Therefore, the indigestible polysaccharides seem to possess many properties related to preserve host health, and the exploring the interaction between polysaccharides and gut microbiota is important for the evaluation the health benefits of natural polysaccharides. Nevertheless, to the best of our knowledge, the digestive behavior and microbial degradation characteristics of *R. tanguticum* polysaccharides (RP) as well as the interaction between RP and gut microbiota have not been completely investigated. Consequently, it is essential to reveal the potential digestive and metabolic behaviors of RP, which is beneficial for promoting the design and production of healthy products based on RP.

In the present study, the possible changes in physicochemical properties of RP during in vitro simulated digestion were measured to reveal its digestive characteristics. Subsequently, the dynamic changes in fermentation characteristics of RP and its regulation effects on gut microbial composition were studied. The findings from the current study could provide useful information on the utilization of RP as a prebiotic supplement in the healthy food and medicine industries.

## Materials and methods

### Materials and reagents

The roots of *Rheum tanguticum* were purchased from a local pharmacy of traditional Chinese medicine at Chengdu, Sichuan, China. α-Amylase (1000 U/mg), pepsin (3000 U/g), bovine serum albumin (BSA), pancreatin (4000 U/g), and inulin were purchased from Sigma-Aldrich (St. Louis, MO, USA) and Aladdin-E (Shanghai, China). In addition, HCl, KCl, NaCl, MgCl_2_(H_2_O)_6_, CaCl_2_(H_2_O)_2_, KH_2_PO_4_, NaHCO_3_, and (NH_4_)_2_CO_3_ were also purchased from Aladdin-E.

### Extraction and isolation of *R. tanguticum* polysaccharides

*R. tanguticum* polysaccharides were extracted by using deep eutectic solvent assisted extraction based on a previously reported method with minor modifications [18]. The deep eutectic solvent was consisted of choline chloride and ethylene glycol (1: 3, molar ratio). Firstly, 10.0 g of sample powders were refluxed with ethanol (80%, *v/v*) for 1 h to remove small substances, such as phenolics and pigments. Then, 180.0 mL of deionized water and 120.0 mL of deep eutectic solvent were added into the extracted residues and the extraction was carried out in a water bath at 95 ℃ for 2 h. After centrifugation, polysaccharides in the extracted solution were isolated by using graded alcohol precipitation and membrane separation. Polysaccharides precipitated with three volumes of ethanol (95%, *v/v*) were collected. Finally, polysaccharides extracted from *R. tanguticum* (RP) were redissolved in deionized water and ultra-filtered (molar mass cutoff, 3.0 kDa) to further remove impurities. The extraction yield of RP was about 8.13%.

### Simulated digestion of *R. tanguticum* polysaccharides

The simulated digestion of RP was carried out as the INFOGEST protocol described with minor modifications [19]. The electrolyte solutions used for simulated salivary fluid (SSF), gastric fluid (SGF), and intestinal fluid (SIF) were prepared based on the literature [19]. In the oral digestion phase, 100.0 mL of SSF electrolyte solution and α-amylase (15,000 U) were added into 100.0 mL of RP solution (20.0 mg/mL), and incubated at 37 ℃ in a water bath shaker. Subsequently, 2.0 mL of the digested samples at the digestive points of 0.25 h and 0.5 h were taken out and placed in boiling water for 5 min. After the oral digestion phase, 150.0 mL of pre-heated SGF electrolyte solution (37 ℃) was mixed with 150.0 mL of the saliva digested sample, and the pH of the mixture was adjusted to 3.0 by using HCl (1 M). Then, pepsin (600 U) was added into the mixture, and incubated at 37 ℃ for 2 h. In the intestinal digestion phase, 150.0 mL of pre-heated SIF electrolyte solution (37 ℃) was mixed with 150.0 mL of the gastric digested sample, and the pH of the mixture was adjusted to 7.0 by adding NaOH (1 M). Then, the pancreatin and bile salts were added into the mixture to reach final concentrations of 100 U/mL and 10 mM, respectively, and incubated at 37 ℃ for 2 h. Moreover, during the gastric and intestinal digestion phases, the digested samples were collected again at 0.5, 1.0, and 2.0 h for further analysis. Finally, the digested mixtures at salivary, saliva-gastric, and saliva-gastrointestinal phases were sequentially treated by 95% (*v/v*) of ethanol (four volumes), ultra-filtered (molar mass cutoff, 3.0 kDa), and freeze dried to obtain RPS, RPG, and RPI at the salivary, saliva-gastric, and saliva-gastrointestinal digestion stages, respectively.

### In vitro fermentation of the indigestible RP (RPI) by human feces

The in vitro fermentation of the indigestible RP (RPI) by human feces was carried out by a previous method with minor modifications [10]. Firstly, 1.0 L of basic fermentation medium was also prepared as described in the literature [10], which consisted of yeast extract, peptone, bile salts, NaCl, CaCl_2_(H_2_O)_2_, NaHCO_3_, K_2_HPO_4_, KH_2_PO_4_, MgSO_4_(H_2_O)_7_, hemin, L-cysteine, resazurin solution, tween 80, and vitamin K. Subsequently, fresh feces were provided by two healthy female volunteers and two healthy male volunteers (ages from 18 to 25), who did not take any antibiotics and had any digestive diseases. 3.0 g of fresh feces were mixed with 30.0 mL of physiological saline (0.9%, *w/v*), and centrifugated (300×*g*) to get the human fecal inoculum. Furthermore, 1.0 g of RPI were dissolved in 100.0 mL of sterilized basic fermentation medium (121 ℃, 20 min), and 9.0 mL of RPI solution was mixed with 1.0 mL of the human fecal inoculum in penicillin bottles for the anaerobic fermentation at 37 ℃ by using an anaerobic chamber (BPN-300CS, Being Instrument, Shanghai, China). At the same time, the basic fermentation medium mixed with inulin was served as the positive control (INULIN group). The negative control (BLANK group) only contained the human fecal inoculum and the basic fermentation medium. Finally, all fermented samples were collected after the in vitro fermentation, and sequentially treated by ethanol (four volumes), ultra-filtered (molar mass cutoff, 3.0 kDa), and freeze dried. The fermented samples of RPI at the fermented time points of 6, 12, 24, and 48 h were named as RPI-6 h, RPI-12 h, RPI-24 h, and RPI-48 h, respectively.

### Measurement of physicochemical properties of *R. tanguticum* polysaccharides during in vitro simulated digestion and fermentation by human feces

#### Measurement of reducing sugar contents (CR), chemical compositions, and fermentabilities

The reducing sugars released from RP after the simulated oral, gastric, and intestinal digestion stages, as well as the in vitro fecal fermentation stages were determined by the 3,5-dinitrosalicylic acid method [11]. The total polysaccharides and uronic acids of RP, RPS, RPG, RPI, RPI-6 h, RPI-12 h, RPI-24 h, and RPI-48 h were investigated through the phenol–sulfuric acid and *m*-hydroxydiphenyl methods, as previously reported [20]. In addition, the fermentabilities (%) of RPI during the fecal fermentation stages in vitro were calculated according to the contents of total sugars and reducing sugars [11].

#### Measurement of monosaccharide compositions and free monosaccharides released

Monosaccharide compositions of RP, RPS, RPG, RPI, RPI-6 h, RPI-12 h, RPI-24 h, and RPI-48 h, as well as free monosaccharides released from RPI during the fermented stages in vitro were determined by Ultimate U3000 LC system (Thermo Fisher Scientific, Waltham, MA, USA) equipped with a phenomenex gemini C18 column (150 mm × 4.6 mm, 5 μm) as previously reported [21]. The mobile phase was a mixture of phosphate buffer solution (0.1 M, pH = 6.7) and acetonitrile (83: 17, *v/v*), and the flow rate was set as 1.0 mL/min.

#### Measurement of molecular weights and FT-IR spectra

Molecular weights of RP, RPS, RPG, RPI, RPI-6 h, RPI-12 h, RPI-24 h, and RPI-48 h were measured by size exclusion chromatography collected with a multi-angle laser light scattering detector and a refractive index detector (Wyatt Technology Co., Santa Barbara, CA, USA), as previously reported [18]. The Shodex OHpak SB-806 M HQ (300 mm × 8.0 mm, i.d.) column was used, and the mobile phase was 0.9% of NaCl solution. Additionally, infrared spectra of RP, RPS, RPG, RPI, RPI-6 h, RPI-12 h, RPI-24 h, and RPI-48 h were measured by a Nicolet iS 10 FT-IR (Thermo Fisher Scientific, Waltham, MA, USA) as previously reported [21]. Besides, the esterification degree (DE) values of RPs were also calculated based on the absorption bands around 1741 cm^−1^ and 1629 cm^−1^.

### Analysis of pH, short chain fatty acids, and gut microbiota during in vitro fermentation by human feces

The pH values of fermented samples at different fermented time points of 0, 6, 12, 24, and 48 h were detected immediately by using a pH meter (RMD-H800, Remond Auto, Shanghai, China). Besides, the contents of short chain fatty acids (SCFAs) generated by colonic microbiota in human feces at different fermented time points of 0, 6, 12, 24, and 48 h were measured by using an Agilent GC system (Agilent Technologies, Santa Clara, CA, USA) equipped with an Agilent HP-INNOWAX column (30 m × 250 μm × 0.25 μm) and a flame ionization detector, as previously reported [22].

Furthermore, the extraction, PCR amplification, purification, and sequencing of the bacterial 16S rRNA genes of the fermented mixture at the fermented time point of 48 h were carried out according to previously reported methods [11, 23]. In addition, the sequencing data were also further processed and analyzed by previously reported methods [11].

### Statistical analysis

All experiments were conducted in triplicate, and the results were showed as mean ± standard deviation. Graphing and statistical analysis were performed by Origin 9.0 software (OriginLab, Northampton, Mass., USA) and SPSS statistics 24.0 software (SPSS 24.0, IBM, Armonk, NY, USA), respectively.

## Results and discussion

### Digestive stabilities of RP at different in vitro simulated digestion stages

#### Digestive stabilities of CR and molecular weight

**Fig. 1:**
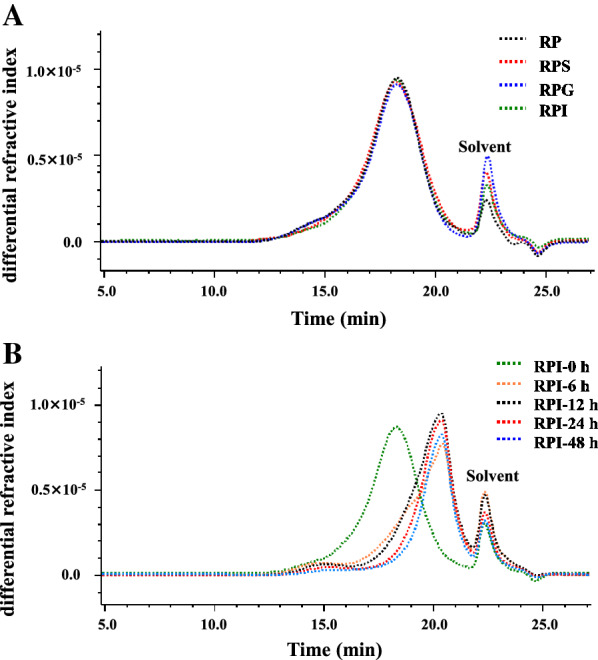
Possible change in SEC profiles of *R. tanguticum* polysaccharides during in vitro digestion (**A**) and human fecal fermentation (**B**). RP, *R. tanguticum* polysaccharides; RPS, RPG, and RPI, RP digested at different digestion stages, including salivary, saliva-gastric, and saliva-gastrointestinal digestions, respectively; RPI-0 h, RPI-6 h, RPI-12 h, RPI-24 h, and RPI-48 h, RPI fermented by human feces at the time points of 0, 6, 12, 24, and 48 h, respectively;

Generally, the α-(1 → 4)-glycosidic bonds of starch or α-polysaccharides can be breakdown by α-amylase which exists in the human saliva. Therefore, the digestive stability of RP during the simulated salivary digestion was studied. As most of non-starch polysaccharides, there were no significant differences in C_R_ during the salivary digestion as shown in Table 1. Furthermore, as displayed in Fig. 1A, the SEC profiles of RP and RPS were the same, and the retention time was also identical. Indeed, the molecular weights of RP and RPS were almost the same, which were determined as 2.571 × 10^5^ Da and 2.575 × 10^4^ Da, suggesting that RP could not be digested by human saliva. Additionally, the C_R_ of RP was also not changed significantly during the gastrointestinal digestion (Table 1). Indeed, no significant changes were observed in SEC profiles of RP, RPG and RPI (Fig. 1A), and their molecular weights were detected to be 2.571 × 10^5^ Da, 2.596 × 10^5^ Da, and 2.588 × 10^5^ Da, respectively, indicating that RP was not sensitive to the gastric fluid and small intestinal fluid. The digestive characteristics of RP were different from several previous studies. For instances, molecular weights of polysaccharides from okra and snow chrysanthemum were partially changed after the simulated digestion, which might be caused by the low pH of gastric fluid and the digestive enzymes existed in the gastrointestinal fluid [10, 11]. Conversely, some similar results have found that polysaccharides from tamarind seed, Fuzhuan brick tea, and *Coralline pilulifera* can be resistant to the gastrointestinal digestion [13, 22, 24]. Therefore, the digestive characteristics of natural polysaccharides were varied by their different sources.

**Table 1:** Possible change in reducing sugar content of *R. tanguticum* polysaccharides during in vitro digestion and human fecal fermentation

Processes	Time (h)	Reducing sugar content (mg/mL)
Salivary digestion	0.25	0.117 ± 0.010 ^a^
	0.5	0.112 ± 0.008 ^a^
Saliva-gastric digestion	0.5	0.156 ± 0.012 ^a^
	1	0.165 ± 0.015 ^a^
	2	0.145 ± 0.010 ^a^
Saliva-gastrointestinal digestion	0.5	0.174 ± 0.009 ^a^
	1	0.171 ± 0.011 ^a^
	2	0.178 ± 0.011 ^a^
Human fecal fermentation	0	0.177 ± 0.003 ^e^
	6	0.778 ± 0.020 ^a^
	12	0.631 ± 0.009 ^b^
	24	0.583 ± 0.017 ^c^
	48	0.496 ± 0.034 ^d^

Values represent mean ± standard deviation, and different superscript lowercase letters indicate significant (*p* < 0.05) in each group

#### Digestive stabilities of chemical composition, compositional monosaccharide, and FT-IR spectrum

The digestive characteristics of chemical compositions, compositional monosaccharides, and FT-IR spectra of RP at different in vitro digestion stages were also measured. As shown in Table 2, the total polysaccharides and total uronic acids of RP were 85.48% ± 0.73% and 38.54% ± 1.31%, respectively, suggesting that carbohydrate was the main component in RP. Notably, no significant variations have been found in chemical compositions of RP during the saliva-gastrointestinal digestion (Table 2). In addition, the compositional monosaccharides of RP, RPS, RPG, and RPI were nearly identical, which were composed of Xyl, GalA, Ara, Gal, Rha, Glc, GlcA, and Man with the similar molar ratios (Table 3), suggesting that compositional monosaccharides of RP were stable under the in vitro digestion conditions. Additionally, the major monosaccharides of RP and its digested samples were determined as GalA, Ara, and Gal. Generally, GalA, Gal, Ara, Rha, and GlcA are typical monosaccharides for homogalacturonan (HG) and rhamnogalacturonan I (RG I), while the typical monosaccharides for hemicelluloses are Man, Glc, and Xyl. Besides, Ara and Gal can also arise from arabinogalactan (AG) [18, 20]. Consequently, a large content of HG and AG as well as a few of RG I and hemicelluloses might present in RP and its digested samples based on the molar ratio of compositional monosaccharides.

**Table 2:** Possible changes in fermentability and chemical composition of *R. tanguticum* polysaccharides during in vitro digestion and human fecal fermentation

	In vitro digestion of RP				In vitro fermentation of RPI			
	RP	RPS	RPG	RPI	RPI-6 h	RPI-12 h	RPI-24 h	RPI-48 h
Fermentability (%)	–	–	–	–	44.62 ± 1.12 ^d^	57.68 ± 1.86 ^c^	68.34 ± 2.18 ^b^	75.49 ± 3.14 ^a^
Total polysaccharides (%)	85.48 ± 0.73 ^a^	85.90 ± 1.13 ^a^	85.98 ± 1.11 ^a^	84.87 ± 0.51 ^a^	48.05 ± 1.29 ^b^	43.44 ± 1.63 ^bc^	40.69 ± 1.25 ^c^	38.71 ± 1.29 ^c^
Total uronic acids (%)	38.54 ± 1.31 ^b^	37.60 ± 2.12 ^b^	38.15 ± 2.03 ^b^	38.26 ± 1.06 ^b^	57.66 ± 1.36 ^a^	60.69 ± 1.30 ^a^	61.21 ± 2.71 ^a^	59.60 ± 1.29 ^a^
Degree of esterification (%)	43.17 ± 0.54 ^a^	45.15 ± 1.39 ^a^	45.02 ± 0.71 ^a^	43.38 ± 1.55 ^a^	15.44 ± 0.61 ^b^	12.43 ± 1.39 ^c^	9.91 ± 0.44 ^c^	5.56 ± 0.59 ^d^

Sample codes were the same as in Fig. 1.

Values represent mean ± standard deviation, and different superscript lowercase letters indicate significant (*p* < 0.05) in each row

**Table 3:** Possible change in compositional monosaccharide of *R. tanguticum* polysaccharides during in vitro digestion and human fecal fermentation

	In vitro digestion of RP				In vitro fermentation of RPI			
	RP	RPS	RPG	RPI	RPI-6 h	RPI-12 h	RPI-24 h	RPI-48 h
Monosaccharides and molar ratios								
Xylose	1.00	1.00	1.00	1.00	1.00	1.00	1.00	1.00
Galacturonic acid	4.03	3.95	3.96	4.01	4.97	5.11	5.19	4.50
Arabinose	3.75	3.72	3.67	3.70	0.50	0.31	0.27	0.25
Galactose	2.63	2.64	2.54	2.58	1.58	1.06	0.97	0.86
Rhamnose	1.01	0.96	0.97	0.95	0.53	0.43	0.39	0.36
Glucose	0.51	0.54	0.53	0.50	0.19	0.18	0.18	0.16
Glucuronic acid	0.44	0.42	0.45	0.43	0.32	0.20	0.16	0.11
Mannose	0.36	0.38	0.37	0.36	0.30	0.29	0.29	0.27

Sample codes were the same as in Fig. 1

**Fig. 2:**
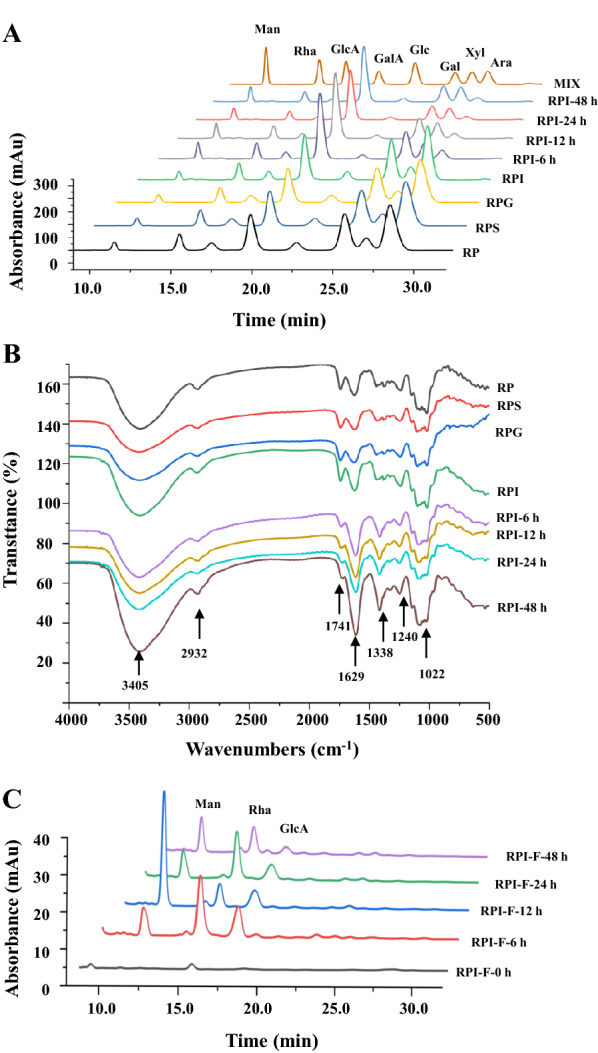
Possible changes in constituent monosaccharides (**A**), FT-IR spectra (**B**), and free monosaccharides released (**C**) of *R. tanguticum* polysaccharides during in vitro digestion and human fecal fermentation. Sample codes were the same as in Fig. 1; RPI-F-0, RPI-F-6, RPI-F-12, RPI-F-24, and RPI-F-48, the supernatants of fermented mixtures of RPI after the in vitro fecal fermentation for 0, 6, 12, 24, and 48 h, respectively. Rha, rhamnose; GalA, galacturonic acid; Ara, arabinose; Gal, galactose; GlcA, glucuronic acid; Glc, glucose; Xyl, xylose;

Moreover, the FT-IR spectra of RP, RPS, RPG, and RPI were investigated to further reveal its digestive characteristics. As shown in Fig. 2B, the FT-IR spectra of RP after the in vitro digestion were similar, indicating that the primarily chemical structure of RP was stable. In addition, the typical signals of acidic polysaccharides, including 3405 cm^−1^, 2932 cm^−1^, 1741 cm^−1^, 1629 cm^−1^, 1338 cm^−1^, 1240 cm^−1^, and 1022 cm^−1^, were measured in RP and its digested samples [18, 20, 21]. Especially, the signals at 1741 cm^−1^ and 1629 cm^−1^ were attributed to the C = O contraction vibration of esterified and free carboxyl groups, respectively. Indeed, the DE values of RP, RPS, RPG, and RPI were calculated based on the intensities of esterified and free carboxyl groups, which ranged from 43.17 to 45.15%. There was no significant difference among them.

### Fecal fermentation characteristics of the indigestible RP (RPI)

#### Dynamic changes in CR, free sugars released, and molecular weight

Generally, non-starch polysaccharides can be hydrolyzed and utilized by gut microorganisms, resulting in the changes of reducing sugar contents [11, 25]. Hence, in order to evaluate the relationship between the depolymerization of polysaccharides and the growth of colonic bacterial during the fermentation by human feces, the reducing sugars released from RPI were detected. As summarized in Table 1, C_R_ in the fermented broth of RPI changed significantly, ranging from 0.177 ± 0.003 mg/mL to 0.496 ± 0.034 mg/mL during in vitro fermentation. More specifically, at the initial fermentation stage (0–6 h), the C_R_ remarkably increased from 0.177 ± 0.003 mg/mL to the maximum value of 0.778 ± 0.020 mg/mL, and then gradually reduced till the end of fermentation process to reach a final value of 0.496 ± 0.034 mg/mL. These results indicated that the degradation of RPI by colonic bacteria was predominant at the initial fermentation stage (0–6 h), and then the utilization of reducing sugars by gut microbiota was faster than that of production with the increasing fermentation time, thereby resulting in the decease of reducing sugars [26]. Similar phenomenon could be also observed in the polysaccharides extracted from *Sargassum fusiforme* and loquat leaves [9, 23]. In addition, the free sugars released from RPI during the fermentation by human feces were also analyzed. As shown in Fig. 2C, three major monosaccharides, including mannose, rhamnose, and glucuronic acid, were detected in the fermentation broth of RPI at the initial stage of fermentation, indicating that RPI was degraded by colonic bacteria. Indeed, at the end stage of fermentation (24–48 h), the responses of mannose, rhamnose, and glucuronic acid were notably decreased, suggesting that the released reducing sugars were utilized by gut bacteria [11].

Moreover, the molecular weight of RPI also changed notably after the in vitro fermentation by human feces. As shown in Fig. 1B, the SEC profiles of RPI shifted from left to right with the increase of retention time. Specifically, the SEC profiles of RPI remarkably shifted to right at the initial stage of fermentation. Indeed, the molecular weights of RPI, RPI-6 h, RPI-12 h, RPI-24 h, and RPI-48 were determined to be 2.588 × 10^5^ Da, 1.447 × 10^5^ Da, 1.263 × 10^5^ Da, 0.927 × 10^5^ Da, and 0.828 × 10^5^ Da, suggesting that RPI was quickly degraded into small fragments by colonic bacteria at the initial stage of fermentation. Besides, taken together with the results of reducing sugars released, it could be presumed that the decline of molecular weight of RPI at different fermented time points was primarily due to the breakdown of its glycosidic bonds. Similar results could also be found in loquat leaf polysaccharides and chrysanthemum polysaccharides during the fermentation by human feces [11, 23].

#### Dynamic changes in fermentability, chemical composition, compositional monosaccharide, and FT-IR spectrum

The fermentabilities and chemical compositions of the indigestible RPI during the fermentation by human feces are presented in Table 2. The fermentabilities of RPI-6 h, RPI-12 h, RPI-24 h, and RPI-48 h were measured to be 44.62%, 57.68%, 68.34%, and 75.49%, indicating that RPI was quickly utilized by gut microbes at the initial stage of fermentation, and almost used at the end stage of fermentation. Besides, the contents of total polysaccharides in RPI, RPI-6 h, RPI-12 h, RPI-24 h, and RPI-48 h remarkably decreased from 84.87% ± 0.51 to 38.71% ± 1.29%. Moreover, the contents of total uronic acids in RPI, RPI-6 h, RPI-12 h, RPI-24 h, and RPI-48 h were increased from 38.26% ± 1.06 to 61.21% ± 2.71%, which might be attribute to the degradation of neutral monosaccharides in RPI during in vitro fermentation process. Furthermore, the dynamic change in compositional monosaccharides was also evaluated to reveal the fermentation characteristic of RPI. Generally, natural polysaccharides can be hydrolyzed by different carbohydrate active enzymes produced by colonic microbes, such as *Bacteroides thetaiotaomicron* and *B. caccae* [14]. Consequently, the monosaccharides of natural polysaccharides can be altered by gut microbes during the fermentation process [23]. As shown in Fig. 2A, the HPLC profiles of monosaccharides in RPI and its fermented samples were consistent. Conversely, the molar ratios of monosaccharides in RPI obviously altered during in vitro fermentation process. Notably, the molar ratios of Ara and Gal in RPI dramatically decreased after the in vitro fermentation for 6 h (Table 3), suggesting that the AG or the branches of RG I that existed in RPI might be preferentially utilized by gut bacteria at the initial stage of fermentation. Additionally, the molar ratios of Rha, GlcA, Ara, Gal, Glc, and Man gradually declined with the increasing fermentation time, suggesting that the RG I and hemicelluloses that existed in RPI might be also utilized by colonic bacteria. Furthermore, the increase of molar ratio of GalA before the fermentation time of 24 h might be related to the notable decrease of other neutral sugars, such as Ara and Gal. Nevertheless, with the increasing fermentation time (24 to 48 h), the molar ratio of GalA was obviously declined, suggesting that the HG that existed in RPI might be utilized. These results suggested that AG might be more easily to be utilized by gut microbiota than that of HG during in vitro fermentation process.

Furthermore, the FT-IR spectra of RPI, RPI-6 h, RPI-12 h, RPI-24 h, and RPI-48 h were also investigated to reveal the fermentation characteristic of RIP. As shown in Fig. 2B, the FT-IR spectra of RPI, RPI-6 h, RPI-12 h, RPI-24 h, and RPI-48 h were similar, indicating that the primary chemical structure of RPI was not changed during in vitro fermentation process. Conversely, the DE value of RPI obviously decreased from 43.38% ± 1.55% to 5.56% ± 0.59% (Table 2), and the degradation of acetyl groups might be attributed to the function of certain enzymes which could be produced by gut microbes [14].

#### Dynamic changes in pH values and SCFAs

The pH value in the colon can be influenced by some acidic metabolites which produced by gut microbes during in vitro fermentation process [27]. Hence, the dynamic change in pH values can serve as a reflection for the degree of fermentation. As shown in Table 4, a slight decline of pH value was found in the BLANK group during in vitro fermentation process. Meanwhile, the pH values of INULIN group and RPI group sharply decreased from 9.11 (0 h) to 6.83 (6 h) and from 7.22 (0 h) to 6.30 (6 h), and then gradually decreased to 5.21 and 5.12 at the end stage of fermentation, respectively. Remarkably, the dynamic change trend in pH values of RPI group was almost identical with that of the content of total polysaccharides in RPI during in vitro fecal fermentation. Similar results have also found in tamarind seed polysaccharides during in vitro fermentation process [13]. Besides, the lower pH values were observed in RPI group at each fermented time point than that of BLANK and INULIN groups, which were much beneficial for gut healthy [9].

**Table 4:** Dynamic variations in pH value and content of SCFAs produced at different fermented time points

Groups	Time (h)	pH	Short-chain fatty acids (mmol/L)						
			Acetic acid	Propionic acid	*i*-Butyric acid	*n*-Butyric acid	*i*-Valeric acid	*n*-Valeric acid	Total
BLANK	0	8.95 ± 0.02	ND	ND	ND	ND	ND	ND	ND
	6	8.75 ± 0.02	6.64 ± 0.02 ^a, B^	ND	ND	ND	ND	1.13 ± 0.22 ^a, A^	7.96 ± 0.20 ^b, C^
	12	8.58 ± 0.03	6.74 ± 0.04 ^a, C^	1.50 ± 0.02 ^a, B^	0.22 ± 0.01 ^a, C^	0.77 ± 0.01 ^a, B^	0.35 ± 0.02 ^a, B^	1.15 ± 0.11 ^a, C^	10.75 ± 0.12 ^a, C^
	24	8.12 ± 0.02	6.80 ± 0.11 ^a, C^	1.54 ± 0.01 ^a, C^	0.22 ± 0.03 ^a, C^	0.84 ± 0.07 ^a, B^	0.33 ± 0.13 ^a, B^	1.20 ± 0.17 ^a, B^	10.95 ± 0.06 ^a, C^
	48	7.86 ± 0.03	6.52 ± 0.07 ^a, C^	1.49 ± 0.01 ^a, C^	0.20 ± 0.03 ^a, C^	0.77 ± 0.01 ^a, B^	0.37 ± 0.02 ^a, B^	1.21 ± 0.06 ^a, B^	10.59 ± 0.03 ^a, C^
INULIN	0	9.11 ± 0.02	ND	ND	ND	ND	ND	ND	ND
	6	7.13 ± 0.02	6.55 ± 0.02 ^c, C^	1.64 ± 0.01 ^b, B^	0.28 ± 0.01 ^b, B^	0.79 ± 0.01 ^a, B^	0.34 ± 0.03 ^a, A^	1.28 ± 0.06 ^a, A^	10.90 ± 0.08 ^d, B^
	12	6.31 ± 0.03	7.37 ± 0.09 ^b, B^	1.65 ± 0.03 ^ab, B^	0.38 ± 0.02 ^b, B^	0.79 ± 0.01 ^a, B^	0.36 ± 0.04 ^a, B^	1.29 ± 0.03 ^a, BC^	11.86 ± 0.09 ^c, B^
	24	5.85 ± 0.02	7.39 ± 0.33 ^b, B^	1.84 ± 0.14 ^a, B^	0.42 ± 0.15 ^b, B^	0.82 ± 0.04 ^a, B^	0.35 ± 0.03 ^a, B^	1.19 ± 0.16 ^a, B^	12.03 ± 0.17 ^b, B^
	48	5.21 ± 0.02	9.43 ± 0.31 ^a, A^	1.82 ± 0.03 ^a, B^	0.81 ± 0.22 ^a, B^	0.83 ± 0.03 ^a, B^	0.37 ± 0.04 ^a, B^	1.13 ± 0.29 ^a, B^	14.42 ± 0.23 ^a, B^
RPI	0	7.22 ± 0.03	ND	ND	ND	ND	ND	ND	ND
	6	6.30 ± 0.02	7.98 ± 0.08 ^b, A^	2.21 ± 0.07 ^b, A^	0.93 ± 0.14 ^c, A^	0.97 ± 0.06 ^c, A^	0.47 ± 0.08 ^c, A^	1.10 ± 0.03 ^c, A^	13.66 ± 0.27 ^c, A^
	12	5.79 ± 0.04	8.23 ± 0.22 ^b, A^	2.35 ± 0.11 ^b, A^	1.02 ± 0.08 ^bc, A^	1.22 ± 0.07 ^b, A^	0.63 ± 0.07 ^b, A^	1.29 ± 0.04 ^b, A^	14.75 ± 0.35 ^b, A^
	24	5.37 ± 0.02	8.55 ± 0.07 ^a, A^	2.56 ± 0.02 ^a, A^	1.20 ± 0.05 ^ab, A^	1.40 ± 0.04 ^a, A^	0.79 ± 0.04 ^a, A^	1.51 ± 0.06 ^a, A^	16.03 ± 0.12 ^a, A^
	48	5.12 ± 0.03	8.74 ± 0.17 ^a, B^	2.55 ± 0.07 ^a, A^	1.24 ± 0.05 ^a, A^	1.47 ± 0.12 ^a, A^	0.86 ± 0.07 ^a, A^	1.59 ± 0.12 ^a, A^	16.45 ± 0.57 ^a, A^

**Fig. 3:**
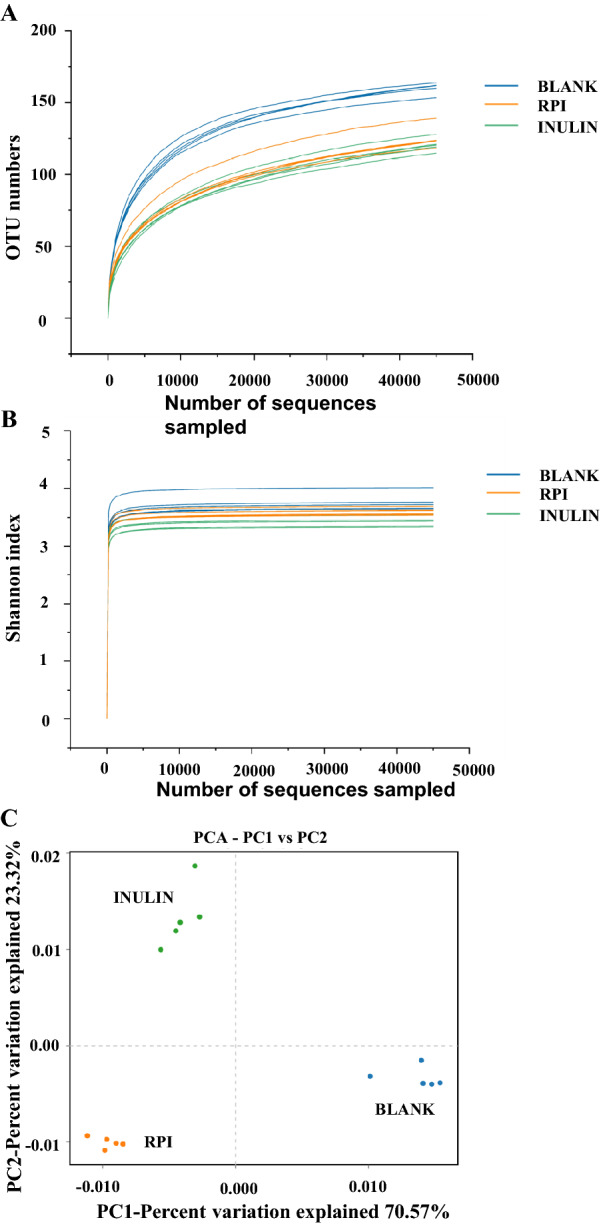
Rarefaction curves (**A**), Shannon indexes (**B**), and principal component analysis (**C**) of gut microbiota of the sample at the fermented time point of 48 h. BLANK, the blank control (no additional carbon source supplement); INULIN, the positive control (INULIN supplement); RPI, the experimental group (RPI supplement)

Sample codes were the same as in Fig. 3; Values represent mean ± standard deviation, and different minuscules showed significant differences among different times (*p* < 0.05) in the same group, while different superscript letters represent significant differences among different groups (*p* < 0.05) at the same time point

*ND* not detected

SCFAs are the major metabolic products generated by colonic microbiota during in vitro fermentation of natural polysaccharides, which play important roles in gut healthy [9]. The concentrations of SCFAs in the BLANK, INULIN, and RPI groups were detected at 0 h, 6 h, 12 h, 24 h, and 48 h of fermentation. As summarized in Table 4, the concentrations of total SCFAs in the BLANK group were detected to be 10.59 ± 0.03 mmol/L after the fermentation for 48 h, while the final concentrations of 14.42 ± 0.23 mmol/L and 16.45 ± 0.57 mmol/L were found in the INULIN and RPI groups, respectively. Besides, compared with the BLANK group, SCFAs generated at different fermented time points in the RPI group significantly increased, especially, the concentrations of acetic acid, propionic acid, *i*-butyric acid, and *n*-butyric acid. Studies showed that most of acetic acids in the gut could be transport to peripheral tissues for providing energy, and the increase of acetic acids may be related to the relatively high abundances of *Bifidobacterium* and *Lactobacillus* [13, 28]. Besides, propionic acid plays an important role in the regulation of cholesterol metabolism and intestinal immune [9, 29]. Butyric acid, as the important energy source of colon cells, has the benefits for preventing intestinal tissues and obesity [29]. As reported, the production of propionic acids and butyric acids might be mainly associated with the fermentation of arabinose and galactose [13, 30]. Thus, the rapid decrease of arabinose and galactose in RPI at the initial stage of fermentation was consistent with the production of propionic acid and butyric acid.

### Effect of the indigestible RPI on the microbial composition

A lot of studies have indicated that intestinal microbes play a key role in human health, and they have the function of fermenting indigestible food, producing metabolites, and maintaining the homeostasis of human body [31]. Indigestible polysaccharides can be fermented by colonic microbiota and regulate the microbial composition which is related to multiple chronic diseases, such as type 2 diabetes mellitus, inflammatory bowel disease, and obesity [13, 32]. Hence, it is necessary to reveal the potential relationship among RPI, intestinal microbes, and microbial metabolites.

In the present study, the alpha diversity was used to reflect the species diversity and abundance in the samples. As shown in Fig. 3A and B, the rarefaction curves and Shannon indexes of three groups gradually tend to be flat, indicating that the sequencing data are reasonable. The lower OTU numbers observed in RPI and INULIN groups indicated the decrease of community diversity when compared with the BLANK group, in consistent with the results from feruloylated oligosaccharides and okra polysaccharides which also decreased the diversity of microbial species [10, 33]. Besides, the principal component analysis (PCA), as the beta diversity analysis method, is used to reflect a distinct clustering of microbial community in different samples [34]. As shown in Fig. 3C, the percent variations for PC1 and PC2 were accounted for 70.57% and 23.32%. Results clearly showed that each group was obviously different from others, indicating that RPI exhibited a different effect on the regulation of the microbial composition.

**Fig. 4:**
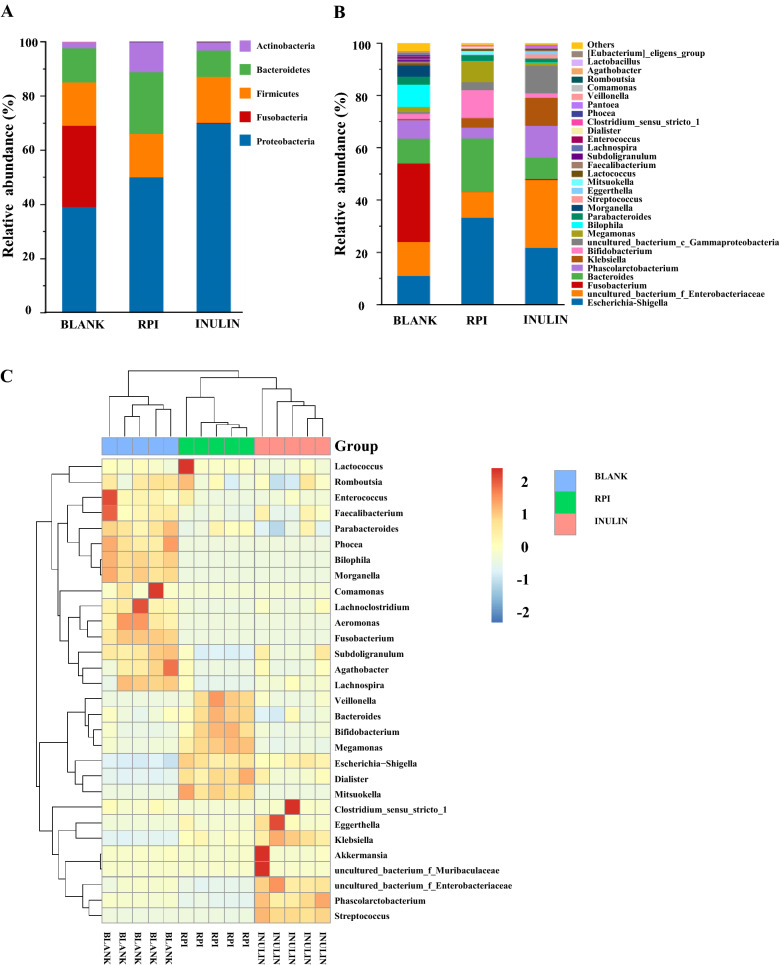
The relative abundance of bacterial community at the phylum level (**A**) and the genus level (**B**) as well as the heat map analysis (**C**) of the relative abundance of bacterial community at the genus level. Sample codes were the same as in Fig. 3

The gut microbial compositions of RPI, BLANK, and INULIN groups at phylum level are exhibited in Fig. 4A. *Proteobacteria*, *Firmicutes*, *Fusobacteria*, and *Bacteroidetes* were the main bacteria in the BLANK group, while *Proteobacteria*, *Firmicutes*, *Bacteroidetes*, and *Actinobacteria* were the predominant gut microbiota in the RPI group. The addition of RPI obviously decreased the relative abundance of *Firmicutes*. Meanwhile, compared with the BLANK group, the ratio of *Firmicutes/Bacteroidetes* in the RPI group was reduced from 1.263 to 0.702 through increasing the relative abundance of *Bacteroidetes*. Reports have found that *Bacteroidetes* was the main colonic bacteria for hydrolyzing indigestible polysaccharides, and producing acetic and propionic acids [24]. Generally, the ratio of *Firmicutes/Bacteroidetes* is positively correlated with obesity, and the reduction of *Firmicutes/Bacteroidetes* ratio indicates the potential weight losing function of RPI [34]. Besides, the relative abundance of *Actinobacteria* also increased in the RPI group and INULIN group, which might be mainly attributed to the increase of *Bifidobacterium*. *Bifidobacterium* is identified as one of the important probiotics in human intestinal tract [11]. *Proteobacteria* increased in INULIN and RPI groups while the growth of *Fusobacteria* was notably inhibited. Usually, *Proteobacteria* can maintain its growth by utilizing low molecular weight carbon sources [11, 12]. Nevertheless, the total abundances of *Proteobacteria* and *Fusobacteria* in the RPI group (50.14%) were obviously lower than that of BLANK group (69.14%). These results suggested that RPI could facilitate intestinal health by promoting the proliferation of several beneficial microbiota and inhibiting the growth of several harmful bacteria.

At the genus level (Fig. 4B), the BLANK group was mainly composed of *Escherichia-Shigella*, *uncultured_bacterium_f_Enterobacteriaceae*, *Fusobacterium*, *Bacteroides*, *Phascolarctobacterium*, and *Bilophila*. Compared with the BLANK group, the RPI increased the relative abundance of several probiotics, such as *Bacteroides*, *Bifidobacterium*, and *Megamonas*. *Bacteroides* is considered as the important gut microbiota to hydrolyze indigestible carbohydrates, and the increase of *Bacteroides* is beneficial for the prevention of obesity [34, 35]. *Bifidobacterium*, a key genus of beneficial microbiota, can hydrolyze macromolecular carbohydrates by producing kinds of glycosidases [27]. Besides, studies suggested that the proliferation of *Megamonas* could suppress the growth of harmful bacteria by competing energy intake [33]. In comparison to healthy people, a relative lower abundance of *Megamonas* was observed in patients with cerebral infarction and ischemia [36]. Furthermore, the RPI decreased the relative abundances of several harmful bacteria, such as *uncultured_bacterium_f_Enterobacteriaceae*, *Bilophila*, and *Fusobacterium*, while promoted the growth of *Escherichia-Shigella* (harmful bacteria). The increase of *Escherichia-Shigella* was also found in previous studies which suggested that *Escherichia-Shigella* could easily utilize the small sugars to maintain its growth in vitro [11, 37]. Furthermore, compared with the BLANK group, the addition of INULIN increased the relative abundance of *Phascolarctobacterium*, which could produce SCFAs [38]. Meanwhile, in the INULIN group, the relative abundance of *Fusobacterium* and *Bilophila* obviously reduced. The total abundances of harmful bacteria in the INULIN group were lower than that of the BLANK group.

The heat map shows the bacteria with the relative abundance above top 30 at the genus level. As shown in Fig. 4C, compared with the BLANK group, an increase of the relative abundances of several beneficial bacteria genus, such as *Dialister*, *Bacteroides*, *Mitsuokella*, *Veillonella*, *Bifidobacterium*, and *Megamonas*, was found in the RPI group. In conclusion, these results indicated that the microbial composition in BLANK, RPI, and INULIN group were significantly different, and the supplementation of RPI could notably change the microbial composition.

## Conclusion

In present study, it was found that RP could pass through the upper gastrointestinal tract safely, and then arrive to the large intestine to be fermented by colonic microbiota. Furthermore, the indigestible RP (RPI) could be easily utilized by colonic microbiota in human feces at the initial stage of fermentation, resulting in the regulation of microbial composition and abundance. Notably, RPI could stimulate the growth of several beneficial bacteria, such as genera *Bacteroides*, *Bifidobacterium*, and *Megamonas*, and promote the generation of SCFAs. The findings from the current study are beneficial to reveal the potential digestive and microbial metabolic characteristics of RP, and RP can be developed and used as a functional food to improve human health via promoting gut health.

## Data Availability

The data used to support the findings of this study are available from the corresponding author upon reasonable request.

## Abbreviations

RP: *Rheum tanguticum* Polysaccharides
RPS: RP collected at the salivary digestion stage
RPG: RP collected at the saliva-gastric digestion stage
RPI: RP collected at the saliva-gastrointestinal digestion stage
RPI-6 h: RPI fermented by human feces at the time point of 6 h
RPI-12 h: RPI fermented by human feces at the time point of 12 h
RPI-24 h: RPI fermented by human feces at the time point of 24 h
RPI-48 h: RPI fermented by human feces at the time point of 48 h
RPI-F-0: The supernatant of the fermented mixture of RPI after the in vitro fecal fermentation for 0 h
RPI-F-6: The supernatant of the fermented mixture of RPI after the in vitro fecal fermentation for 6 h
RPI-F-12: The supernatant of the fermented mixture of RPI after the in vitro fecal fermentation for 12 h
RPI-F-24: The supernatant of the fermented mixture of RPI after the in vitro fecal fermentation for 24 h
RPI-F-48: The supernatant of the fermented mixture of RPI after the in vitro fecal fermentation for 48 h
SCFAs: Short chain fatty acids
SSF: Simulated salivary fluid
SGF: Simulated gastric fluid
SIF: Simulated intestinal fluid
